# The Chemical-Mineralogical Characterization of Recycled Concrete Aggregates from Different Sources and Their Potential Reactions in Asphalt Mixtures

**DOI:** 10.3390/ma13245592

**Published:** 2020-12-08

**Authors:** Edgar H. Sánchez-Cotte, Carlos Albeiro Pacheco-Bustos, Ana Fonseca, Yaneth Pineda Triana, Ronald Mercado, Julián Yepes-Martínez, Ricardo Gabriel Lagares Espinoza

**Affiliations:** 1 Facultad Tecnológica, Universidad Distrital Francisco José de Caldas, Cl. 68D Bis A Sur, Bogotá 49F-70, Colombia; esanchez@udistrital.edu.co; 2 División de Ingeniería, Universidad del Norte-km 5 Vía Puerto Colombia, Barranquilla, Colombia; cbustosa@uninorte.edu.co (C.A.P.-B.); fonsecama@uninorte.edu.co (A.F.); lagaresr@uninorte.edu.co (R.G.L.E.); 3 Facultad de Ingeniería, Universidad Pedagógica y Tecnológica de Colombia, Avenida Central del Norte 39-115, Tunja 150003, Colombia; yaneth.pineda@uptc.edu.co; 4 Grupo de Investigación en Fenómenos Interfaciales, Reología y Simulación de Transporte (FIRST), Universidad Industrial de Santander, Cl. 9 Bucaramanga, Cra 27 Santander, Bucaramanga, Colombia; ramerca@uis.edu.co

**Keywords:** recycled concrete aggregate (RCA), natural aggregates (NA), chemical characterization

## Abstract

The incorporation of a recycled concrete aggregate (RCA) as a replacement of natural aggregates (NA) in road construction has been the subject of recent research. This tendency promotes sustainability, but its use depends mainly on the final product’s properties, such as chemical stability. This study evaluates the physical and chemical properties of RCAs from two different sources in comparison with the performance of NA. One RCA was obtained from the demolition of a building (recycled concrete aggregate of a building—RCAB) and another RCA from the rehabilitation of a Portland cement concrete pavement (recycled concrete aggregate from a pavement—RCAP). Characterization techniques such as X-ray fluorescence (XRF), X-ray diffraction (XRD), UV spectroscopy, and atomic absorption spectrometry were used to evaluate the RCAs’ coarse fractions for chemical potential effects on asphalt mixtures. NA was replaced with RCA at 15%, 30%, and 45% for each size of the coarse fractions (retained 19.0, 12.5, 9.5, and 4.75 sieves in mm). The mineralogical characterization results indicated the presence of quartz (SiO_2_) and calcite (CaCO_3_) as the most significant constituents of the aggregates. XFR showed that RCAs have lower levels of CaO and Al_2_O_3_ concerning NA. Potential reactions in asphalt mixtures by nitration, sulfonation, amination of organic compounds, and reactions by alkaline activation in the aggregates were discarded due to the minimum concentration of components such as NO_2_, (–SO_3_H), (–SO_2_Cl), and (Na) in the aggregates. Finally, this research concludes that studied RCAs might be used as replacements of coarse aggregate in asphalt mixtures since chemical properties do not affect the overall chemical stability of the asphalt mixture.

## 1. Introduction

**Figure 1:**
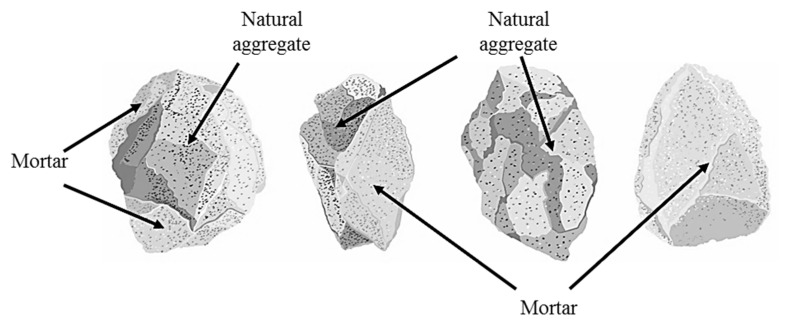
Typical conformations of the recycled concrete aggregate (RCA).

Waste generation during the execution of construction activities (construction and demolition waste—CDW) has prompted researchers from several countries to develop projects aimed at making construction waste reuse viable [1,2,3,4,5]. A material that can form part of this waste is Portland cement concrete (PCC), which, when crushed, is transformed into a recycled concrete aggregate (RCA). Re-incorporating an RCA into the productive cycle reduces its final disposal volume and the exploitation of quarry to obtain stone materials. Waste minimization complies with the principles of efficiency and Sustainable Development Goals 7, 12, and 13, promulgated by the United Nations—the UN. However, RCAs differ in some physical, chemical, and mechanical properties in relation to natural aggregates (NA), mostly due to the presence of bonded mortar [6]. The presence of mortar, which originates at a weaker interfacial zone between the binder and RCA, increases the porosity and water absorption of RCAs, and also reduces their strength and mechanical performance when used in the production of asphalt mixtures [7]. In Figure 1, the different conformations of RCA are shown, taking into account the fractionation due to the crushing process. These conformations indicate that the structure of the RCA is heterogeneous since it can be made up of natural aggregate, mortar, or a combination of both.

Standard physical and mechanical laboratory tests for checking the quality of NA are used to evaluate RCAs and their potential performances in asphalt mixtures [8,9]. However, the study of the chemical and mineralogical properties of RCAs provides further information about the phenomena occurring in mixtures of RCAs and other materials [10]. Besides, the chemical laboratory tests evaluate the presence of undesirable substances or elements in these aggregates, such as chlorides, sulfates, carbonates, and the contaminants absorbed from their original project sources [6,8,9].

The chemical and mineralogical compositions of RCAs are varied and do not follow a general pattern in terms of its elements, compounds, and concentrations due to the various RCA sources and the different dosages of their original concrete components [6,8,11]. Further, the chemical composition of RCAs has not been researched extensively [6,8], which makes it challenging to achieve a standardized procedure for their use [12]. On the other hand, studies highlight the importance of applying image analysis to determine aspects such as residual mortar after the use of RCAs in concrete mixtures, estimation of porosity distribution, and degradation characteristics within concrete [13,14,15].

Cement is a constituent of PCC. Cement has a significant effect on the chemical composition of an RCA because cement is present in the attached mortar, which is considered the weakest part of PCC. Cement is produced from mineral materials such as limestone and gypsum, an alumina base, and silica naturally found as clay or shale [16]. Limestone, which is the cement base, is composed of 60% calcium carbonate (CaCO_3_) and the rest (40%) include clay, silica, and dolomite. However, RCAs can contain salts formed from potassium (K) and cobalt (Co), and potassium (K) and iron (Fe), which can cause aging in asphalt pavements, as they can oxidize asphalt cement [17]. Based on the chemical and mineralogical composition, the following concepts from previous investigations explain the effects of RCAs on asphalt mixtures: (a) The presence of salts in the aggregate indicates that there is a higher electrical conductivity. Therefore, a lower electrical resistivity makes the aggregate susceptible to the penetration of chloride ions when embedded in concrete [16,17]. (b) When there are manganese salts in the aggregates, and the aggregates enter into contact with air, an oxidizing effect occurs in the asphalt cement, generating premature aging [17]. (c) The presence of iron oxides can cause aging and deterioration of the asphalt pavement when catalytic reactions are generated in the asphalt cement [18,19]. (d) The presence of magnesium in a mineral form called periclase can cause a volume increase in contact with water, owing to its hygroscopic property, causing stresses on the internal structure [20]. (e) The carbonates of calcium and magnesium (dolomites) are prone to generate an alkali-carbonate expansive reaction through the dedolomitization process. This process forms brucite Mg(OH)_2_ and regenerates the alkaline hydroxide in the concrete. Generally, Mg(OH)_2_ formation weakens the cement paste junction and the porous zone in the periphery of the aggregate, which generates hygroscopic characteristics and affects its physical properties. (f) The regeneration of the alkali ion (OH)^−^ in a solution makes dedolomitization a continuous process that can affect the recycled aggregates, since it produces an increase in volume and possible generation of fissures [21], hence the need to incorporate aggregates with low alkaline reactivity [22]. (g) The carbonation process occurs when the concrete is exposed to atmospheric contaminants, which favors the appearance of microfractures. It reduces the material strength due to the cycles of crystallization and carbonation expansion related to the alternate wetting and drying of the material [23]. (h) The different conditions and environmental factors to which the RCA concrete is exposed to during its useful life are other factors that can cause weakening and increase porosity. These exposures tend to favor chloride and sulfate attacks [16]. (i) A property of the RCA that affects the adherence behavior with asphalt cement is pH [24,25]. Adhesion is favored at a higher pH (alkaline), as is the case for limestone aggregates. Adhesion decreases when the aggregates are acidic or neutral, such as aggregates containing aluminates and silica (i.e., basalts or granites).

Based on the information presented above, Table 1 summarizes the results of physical property tests, and Table 2 summarizes the results of XRF tests reported by several researchers. The results of these tests depend on the RCA sources.

To understand more about the chemical and physical behavior of RCAs in asphalt, it was necessary to carry out some laboratory tests to identify their relationships.

RCA has been investigated in various proportions, sizes, and fractions as a replacement for NA in asphalt mixtures. However, the performance results obtained for the mixtures do not show a tendency to affirm acceptable behavior when RCA is included. The design characteristics of the mixture containing recycled concrete, and the mineralogy of its aggregates, mark the properties of RCAs such as density, absorption, and wear [35]. The replacement ratio affects the behavior of the resulting concrete. In general, the aggregates are subjected to physical and mechanical tests to evaluate the quality of the aggregates used in asphalt mixtures. However, as mentioned earlier, there are chemical and mineralogical characteristics that affect these mixtures.

Reference [9], reported the mineralogy of RCA in different areas of Portugal, with predominant results of quartz, calcite, K feldspar, and sodium feldspar; they also identified a high concentration of polluting species such as chlorides and sulfates that were not suitable for RCAs to be reused [9].

**Figure 2:**
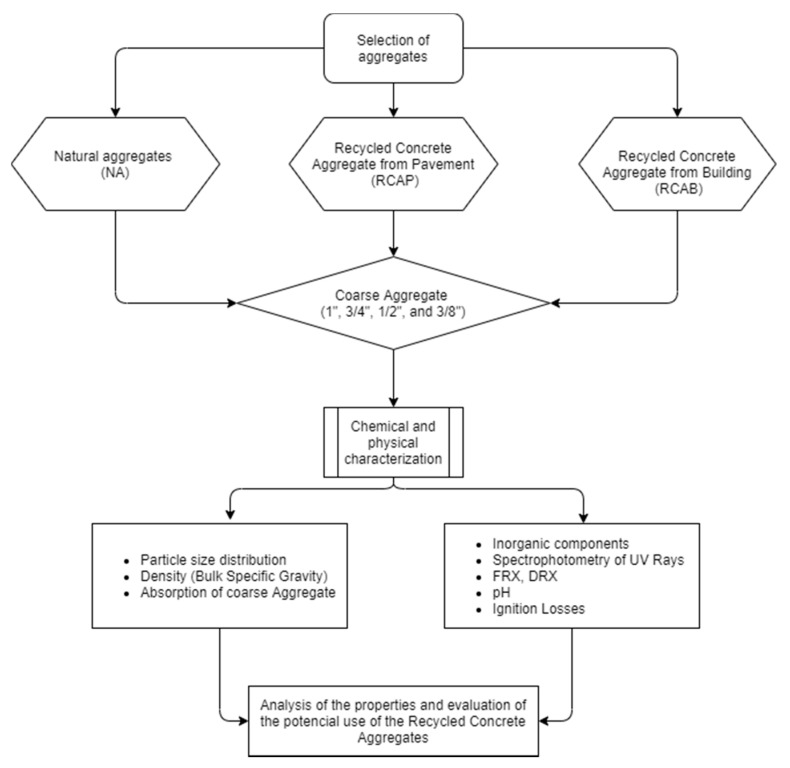
Flow chart of the study plan.

Reference [33] provided important data to be considered in the development of correct recycling strategies, based on the chemical and mineralogical compositions of different granulometric fractions of RCAs [8]. Reference [6] identified that replacements up to 30% of coarse RCAs in concrete mixtures do not influence the chemical composition in terms of the main components (SiO_2_, Al_2_O_3_, and CaO), revealing a direct correlation between the chemical composition of solids and the leaching of ions through ICP-AE analysis of eluates. That highlighted the importance of a correct characterization of the leaching behavior of these new materials [33]. Given the importance of the chemical characterization of RCAs, the present study aimed to analyze the chemical properties of RCAs from two demolition sources in an area of the Colombian Caribbean and a source of NA (recycled concrete aggregate of a building—RCAB; recycled concrete aggregate from a pavement—RCAP; natural aggregate—NA) to evaluate their potential influences on the behavior of asphalt mixtures. This should make a contribution to the knowledge about the characterization of these concrete residues according to the area to which they are exposed within their useful lives. Chemical stability was analyzed from the concentrations of the reactants that could be involved in potential asphalt–mineral reactions. The study presents an alternative approach that involves the chemical properties of RCAs as a criterion for defining the behavior of asphalt mixtures. The samples were evaluated by different techniques to identify the properties of the NA and RCAs (RCAP and RCAB). The characterization tests carried out were: X-ray fluorescence spectrometry (XRF), diffraction spectrometry, X-ray diffraction (XRD), soluble ion analysis by UV–Vis spectrophotometry, atomic absorption analysis, loss on ignition (LOI), determination of mass, percentage of organic impurities, and pH. Figure 2 shows the flow chart used to characterize, analyze, and evaluate the properties of the NA, RCAB, and RCAP aggregates for use in asphalt mixtures.

## 2. Materials and Methods

### 2.1. Materials

The aggregates were obtained from three different sources: a natural commercial aggregate of the research area (NA), an aggregate from the demolition of a concrete road (RCAP), and an aggregate from the demolition of a building (RCAB). The natural aggregate (NA) is representative of the typical materials used in the Department of Atlántico (municipality of Arroyo de Piedra), Barranquilla, Colombia. The RCAP was sourced from the reconstruction of the PCC pavement of the access road to the area port of Barranquilla, Avenida Hamburgo. The RCAB originated from the demolition of the Tomás Arrieta Stadium, in Barranquilla, Colombia. The crushing was carried out at a company plant dedicated to the exploitation and commercialization of stone aggregates located in the area of study. The grain size distribution of all the aggregates followed the Colombian technical specifications for highway construction [36] for an HMA-25 (nominal maximum aggregate size—NMAS—of 25 mm). The analyzed aggregates were coarse fractions. To evaluate the physical, chemical, and mineralogical properties of the aggregates, the following tests were carried out.

### 2.2. Physical Characterization

#### 2.2.1. Particle Size Distribution

One of the fundamental characteristics of aggregates to be used in asphalt mixtures is related to the distribution of aggregate particle sizes. The granulometric range to be obtained by combining the different fractions must be selected to avoid segregation, and thus, guarantees the levels of compaction and strength required. RCAs replace NA in different percentages according to the characteristics of the aggregates. It is, therefore, necessary to generate combinations for optimizing the substitution percentages and obtaining the best functional behavior. Table 3 shows the size distribution ranges of the aggregates (NA and RCA).

**Figure 3:**
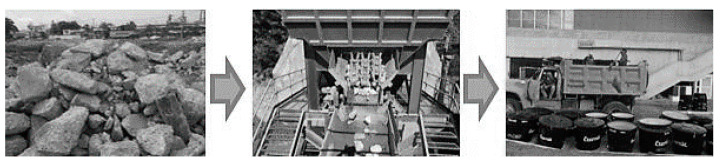
Crushing processes to obtain the RCA.

**Figure 4:**
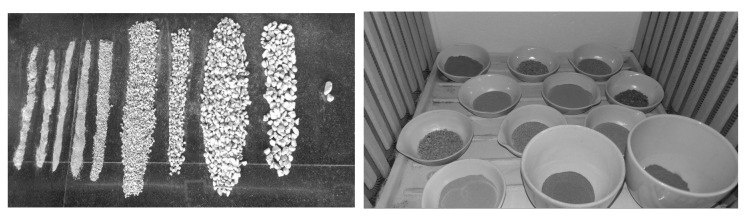
Crushed size distribution of the recycled concrete aggregate from a pavement.

The size obtained after the crushing process is related to the type and technology of the machinery used, and the aggregate graduation parameter. Figure 3 shows the processes of collecting, crushing, sifting, and storing used in obtaining the aggregates (NA, RCAP, and RCAB). Figure 4 displays the size distribution of the aggregates.

In Table 4, it is possible to observe the percentage distribution for each material used in the granulometric spindle with NA and the mixtures, including RCAs, in different percentages of coarse and fine sizes (mass replacement).

#### 2.2.2. Density, Relative Density (Specific Gravity), and Absorption of Coarse Aggregate

The technique used to establish the mass ratio (dry to the furnace, saturated superficially dry, and submerged), and to measure the relative density and absorption of the coarse aggregate followed the ASTM C 127-07 standard, which indicates how to measure the masses of the aggregates in the mentioned conditions, and how to establish the corresponding relationships between them.

### 2.3. Chemical Characterization

#### 2.3.1. Ignition losses

The loss ignition technique was used to determine the organic and inorganic fractions of the RCAs [16]: 550 °C for the organic matter content and 1000 °C for the inorganic fractions of the samples. The samples were prepared according to NTC-ISO 11464 requirements. This methodology involves different stages: 1. Selection and sample preparation. 2. Drying of the sample according to ASTM D 2216-10 (method A) for 24–36 h in a JeioTech Model 55 L oven. 3. Ignition of the sample at 550 °C for 4 h in a Vulcan S, Model 550 muffle, in which the organic matter was gasified, resulting in a difference from the original weight. 4. Final ignition continued at 1000 °C for 2 h more, in which the inorganic carbon was gasified, and the new weight and difference were determined using a Precisa Model 180A analytical balance. 

#### 2.3.2. pH

pH is essential because it has a direct effect on the reaction with the asphalt, altering the ideal conditions. NTC-ISO 11464 and NTC 5264-2008 (method A) standards were followed to determine the pH values. Samples weighing 10.0 g each were diluted in a 1.0:1.2 ratio after obtaining dense mixtures with 1.0: 1.0 ratios; the samples were manual mixed, and two replicates were made for the trials. For the tests, 25 mL beakers were used. A multi-parameter equipment WTW brand model Multi 3420 was used to obtain the conductivity, pH, and temperature measurement in compliance with NTC 5264-2008 standard.

The chemical composition of the RCA was determined by X-ray fluorescence (XRF) of the coarse fraction particle sizes (retained 19.0, 12.5, 9.5, and 4.75 sieves in mm), which were crushed until they passed through sieve #200 (<0.075 mm). Semi-quantitative and semi-qualitative analysis were carried out using a PANalytical Minipal2 spectrometer, with a source of rhodium radiation and energy parameters of 1 to 30 keV.

The elemental content of the RCA was determined by atomic absorption spectrometry, using samples having sizes less than 44 μm (Sieve #200). The samples were treated by digestion with an acid mixture according to EPA 3051A in a microwave digester Mars 6 under standard methods SM: 3030E, 3111B, 3111D for elemental analysis in Shimadzu 7000 flame atomic absorption spectrometer.

The concentrations of SO_4_^2−^, Cl^−^, NO_3_^−^, and PO_4_^3−^ ions present in the RCA were determined by the turbidimetric method (EPA 9038), standard method 4500-Cl-B, cadmium reduction (EPA-353.3), and the ascorbic acid method (EPA 365.1), respectively. Subsequently, the Hach DR 6000 visible UV spectrophotometer was read.

### 2.4. Mineralogical Characterization

The mineralogical composition of each RCA was determined by X-ray diffraction (XRD), with 10.0 g samples, which were crushed until they passed through sieve #200 (<0.075 mm). Effervescence tests and magnetic field tests were carried out. A Phillips x’pert pro PANalytical diffractometer was used with CuKa radiation (40 kV and 35 mA) at 2 h-angular within a range of 10°–80° with a 0.5 s/step sweep speed. The analysis was carried out through the X’pert High Score plus^®^ program, taking the diffraction patterns of the Inorganic Crystal Structure Database (ICSD) as the reference.

## 3. Results and Discussion

### 3.1. Physical Characterization

#### 3.1.1. Particle Size Distribution

**Figure 5:**
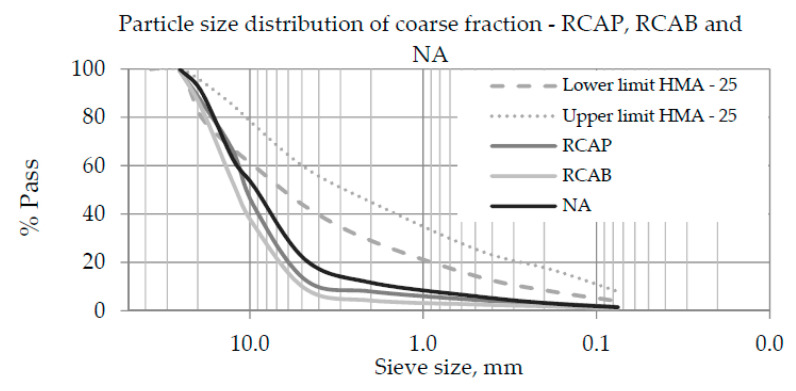
Particle size distribution curves of the coarse fractions of the recycled concrete aggregate from a pavement, aggregate from the demolition of a building (RCAB), and natural aggregate (NA).

The particle size distribution of coarse aggregate materials plays an important role in the design and performance of asphalt mixtures. The coarse fraction was defined considering that, in general, the fine fraction of the RCA has higher absorption and lower density compared to the coarse fraction [28], since the optimum asphalt content (OAC) will increase to a lesser extent [12]. Figure 5 displays the particle size-distribution curves of the coarse fractions of NA, RCAP, and RCAB. The particle sizes of the three materials were not within the limits of HMA-25, which indicates that the NA and RCAs should be combined with the fine fraction and filler to meet the specifications.

#### 3.1.2. Density and Absorption

Although the regulations do not establish maximum values of absorption or minimum density, it is convenient to avoid highly absorbent aggregates in asphalt mixtures [37]. Some authors consider highly absorbing aggregates as those that exceed 5% water absorption; for instance: in Belgium, the maximum absorption allowed for coarse aggregate is 10%, while in Japan, it is 7% [38]. Table 5 presents the values of the density and water absorption of the aggregates within the coarse size fractions for NA, RCAP, and RCAB. When the aggregate size decreases, its absorption increases due to the larger surface area, and its specific gravity decreases owing to the smaller quantity of mortar as a consequence of the crushing. It is also observed that RCA has higher absorption and lower density than NA; the mixtures manufactured with RCA will have a higher OAC (optimum asphalt content).

### 3.2. Chemical Characterization

#### 3.2.1. Loss of Ignition (550 °C and 1000 °C)

**Figure 6:**
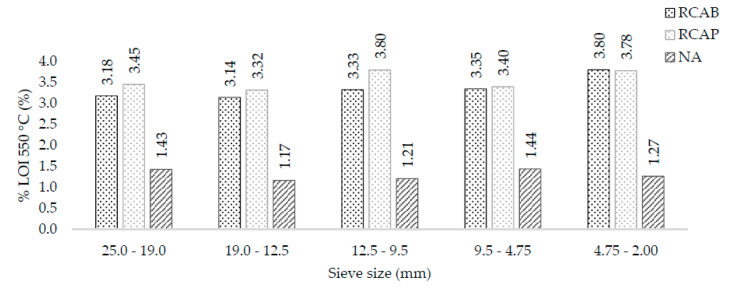
LOI550 °C of NA, RCAP, and RCAB by size.

The coarse fractions of the aggregates were characterized based on their weight loss when subjected to 550 °C and 1000 °C. These ignition losses were directly related to the organic matter content and thermal decomposition of the aggregates. When the test was performed at 550 °C, the moisture evaporated, and the organic material was volatilized. Figure 6 shows the mass loss at 550 °C for the 19.0 mm, 12.5 mm, 9.5 mm, 4.75 mm, and 2.0 mm sieve sizes. It can be noticed that recycled aggregates experienced greater weight loss than the natural aggregate. We affirm that the RCAB and the RCAP have higher contents of volatile organic material (VOM). This higher content was directly related to the longer environmental exposure time of the recycled aggregates. As for the RCAB, the VOM and humidity contents ranged from 3.18% to 3.80%, with a tendency to increase when the particle size decreased. For RCAP, these contents ranged between 3.45% and 3.78%. By comparing these results with those of the natural aggregate, the amount of VOM was about 1% to 2% lower in NA.

**Figure 7:**
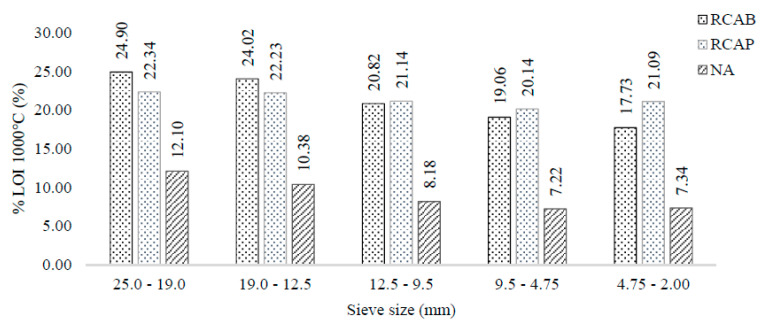
LOI 1000 °C of NA, RCAP, and RCAB by size.

Ignition losses at 1000 °C initially involved the evaporation of moisture from the aggregates, followed by the vaporization of VOM. Above 900 °C, decomposition reactions of the carbonates present in the aggregates were produced. This test gives an idea of the content of dolomite and other carbonate minerals, such as ankerite. Figure 7 presents the mass loss at 1000 °C in each of the 19.0, 12.5, 9.5, 4.75, and 2.0 mm sieve sizes. The ignition loss at 1000 °C was around 20% to 25% for the RCA, and between 7% and 8% for the NA. This difference reflects on the origins of both materials. Regardless of whether the recycled aggregates had been exposed to environmental effects, their origin appeared to be different from that of the NA. It is possible to say that the content of the carbonated minerals (mainly dolomite) was higher in the RCAs, which can be evidenced by the XRD tests. Besides, the mineralogy of the RCA seemed to be similar, presenting equivalent carbonate contents of calcium, magnesium, and manganese, whereas the NA had higher silicate content. Therefore, the results indicate that the chemical stability of RCA will not be compromised, although from the mineralogical point of view, the difference may imply a change in mechanical behavior.

#### 3.2.2. pH of NA, RCAP, and RCAB

**Figure 8:**
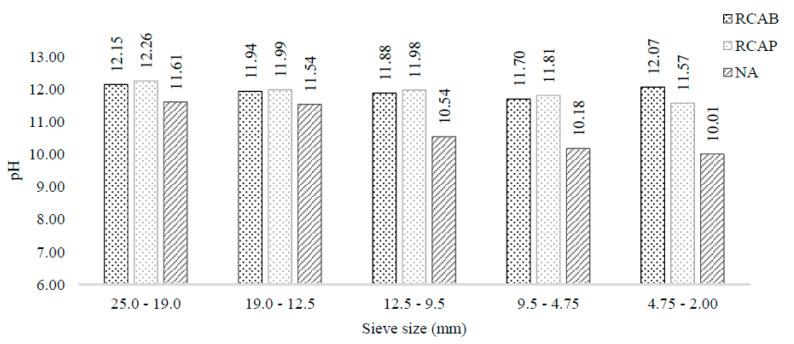
pH of NA, RCAP, and RCAB by particle size.

As seen in Figure 8, both the NA and RCA indicated alkaline pH values. However, RCAs did not exhibit a statistically significant difference with 95% confidence in the pH as the particle size changed, whereas the NA tended to have lower pH with the decrease in size.

These results agree with what is expected for a natural aggregate and can be interpreted as the absence of a calcareous binder in these small-sized natural aggregates. The natural aggregate does not contain binders such as cement, which may explain the tendency towards neutral pH of the solutions that come in contact with particles below 9.5 to 4.75 mm.

The results suggest that larger natural aggregates (above the 4.75 mm) have water-soluble alkaline compounds beyond the carbonates from minerals such as limestone. Thus, an RCA could influence the asphalt mixture behavior and reduce the adhesion between the aggregates and asphalt.

However, the obtained results suggest that coarse RCAs have water-soluble alkaline components beyond mineral carbonates, such as limestone and dolomite. Therefore, those constituents can influence the behavior of asphalt mixtures and reduce their adhesion in the mixture.

#### 3.2.3. X-ray Fluorescence (XRF)

Table 6 shows the chemical compositions of NA, RCAP, and RCAB obtained by XRF analysis expressed in weight (%). The main oxides were Al_2_O_3_ (6.85–12.00%), SiO_2_ (32.13–49.88%), and CaO (20.85–43.10%). The SO_3_ content was less than 0.75% for the RCAs, which is critical to avoid the formation of nitration reactions.

**Figure 9:**
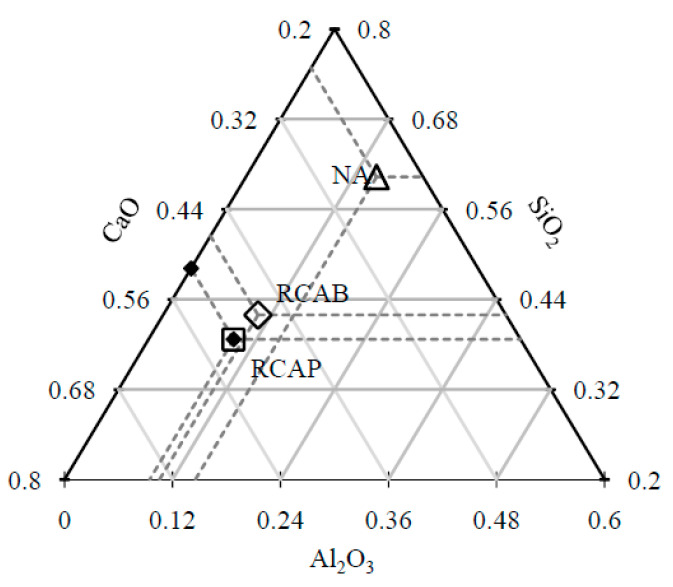
Positions of the NA, RCAB, and RCAP on the CaO–SiO_2_–Al_2_O_3_ ternary diagram.

Figure 9 shows the representation of the CaO–Al_2_O_3_–SiO_2_ system as a ternary phase diagram for the proportions of these compounds present in the coarse aggregates. NA has high SiO_2_ content and low levels of CaO and Al_2_O_3_, whereas RCAP and RCAB have similar CaO and SiO_2_ contents but low Al_2_O_3_ content. The closeness of RCAB and RCAP in Figure 9 indicates similarity in their composition, which is related to the initial structure of the concrete from where they were sourced.

According to the results of Table 6 and Figure 9, the increases in CaO and MgO contents in the RCAs with respect to the NA can be attributed: (a) to the origins of the RCAs and (b) samples of calcite from cement mortars or rubble from bricks, a product of demolition. Likewise, the SiO_2_ contents of the RCAs in a lower proportion than the NA, show a more basic and therefore more hydrophobic tendency, which could favor their use in asphalt mixtures [39].

**Figure 10:**
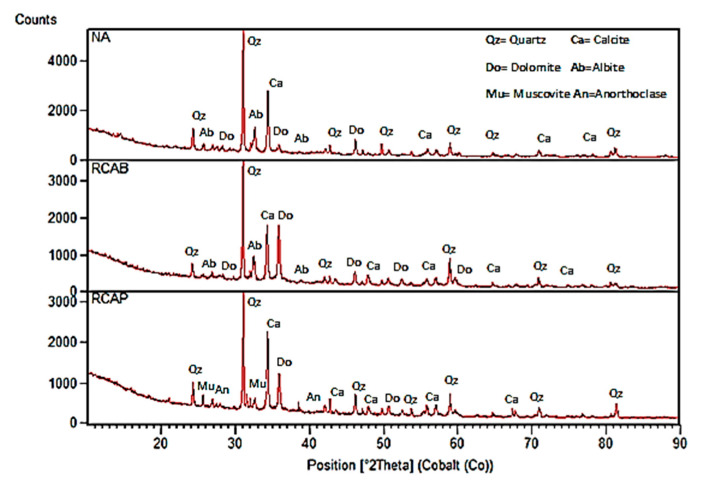
XRD patterns of NA, RCAP, and RCAB.

Figure 10 shows the chemical analysis patterns for the NA, RCAB, and RCAP samples of the semi-quantitative type presented as predominant peaks of quartz mineral (SiO_2_) and calcite (CaCO_3_). The NA and RCAB had sodium silico-aluminates (NaAlSi_3_O_8_) called albite, belonging to the family of feldspars, with peaks of lower intensity.

RCAB had a higher content of dolomite [CaMg(CO_3_)_2_] (34.3%) compared to RCAP (27.3%). This higher content can be attributed to the nature of the aggregate and the cement composition used in the concrete manufacturing.

In the case of RCAP, both muscovite KAl_2_(AlSi_3_) O_10_(OH)_2_ and anorthoclase (6SiO_2_Al_2_O_3_(K, Na)_2_O), were present in proportions of less than 5%. Similarly, the presence of pyroxenes or steatite was demonstrated, which can be attributed to traces of pozzolans in the residues.

**Figure 11:**
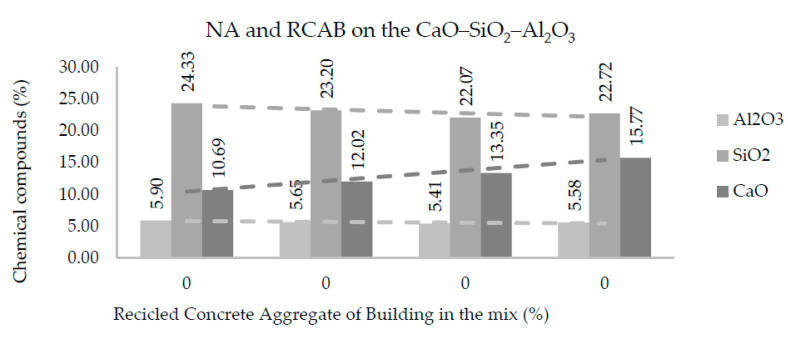
Effects of RCAB percentage on Al_2_O_3_, SiO_2_, and CaO contents in the mixtures of aggregates.

**Figure 12:**
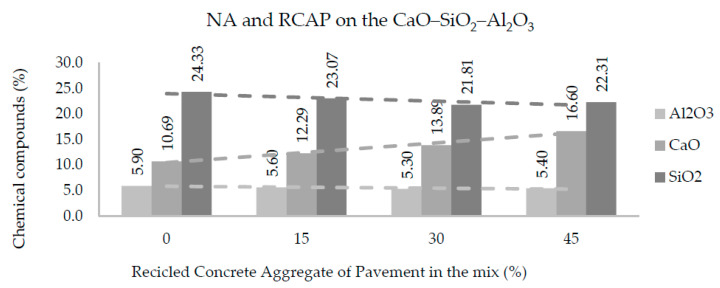
Effects of RCAP percentage on Al_2_O_3_, SiO_2_, and CaO contents in the mixtures of aggregates.

Figure 11 and Figure 12 show the amounts of CaO, SiO_2_, and Al_2_O_3_ in each of the mixtures produced with NA, RCAB, and RCAP. A more significant presence of silicon dioxide (SiO_2_) was observed when there was no RCA mixture (RCAP and RCAB). The SiO_2_ reduced from 24.3% to 22.7% such that the percentage of RCAB increased, and between 24.3% to 22.3% for the increase in RCAP content, because of the lower presence of mortar. Calcium oxide (CaO) increased with recycled aggregate content in the mixture, while the aluminum oxide (Al_2_O_3_) did not show any significant difference.

Table 7 shows the results obtained from the ions dissolved in the NA and RCA using visible ultraviolet spectroscopy. The chloride content in the RCA considerably exceeded that of NA, with a more significant presence in RCAP that may be associated with its origin corresponding to a road located near the coast. Moreover, the chloride content in the RCA was higher for the coarse fraction (25.0–9.5 mm), which can be attributed to a larger exposed surface area of mortar adhered to Cl^−^ present. On the other hand, the sulfate content in the natural aggregate was higher than for RCAs for sizes 25.0–19.0, 19.0–12.5, and 12.5–9.5 mm, which can be associated with the aggregate source. The sulfate content in RCAB was higher for all the fractions compared to RCAP, which may cause susceptibility to degradation attributable to the expansion and cracking in the concrete of corresponding asphalt mixtures [40]. The presence of nitrates in both NA and RCAs were similar. However, the highest content was reported for RCAP for the 25.0–19.0 mm, which may be associated with group concentrations NO_3_^−^ of surface water in contact with the material in service.

To determine the environmental impact related to ionic leaching in the asphalt mixtures, an analysis was performed using atomic absorption spectrometry for the different fractions of the three aggregates. In Table 8, Si and Ca are the most representative elements due to the mineralogical nature of the aggregates. In addition, chemically analyzed RCA samples contained different types of sand and cement, whose main compositions were CaO and SiO_2_. The heavy metal values were insignificant in the leaching process. For example, the lead content did not exceed 0.3 mg/L. This condition means that RCAs will not become sources of environmental pollution, a vital criterion to be subsequently used without toxic risk. The presence of mercury in one of the fractions (9.5–4.75 mm) of RCAB with a value of 11.56 mg/L is explained by the existence of mercury in soils. Reactions with some complex compounds in organic matter (especially fulvic and humic acids) and soil clays cause the mercury to remain for some time. These compounds can reach the RCAs by the exploitation of river sands or soils contaminated with mercury [34]. In the atmosphere, mercury can be found due to natural causes (such as volcanic eruptions, volatilization of aquatic and marine environments) or anthropogenic factors (industrial processes). Depending on the way mercury is found, it can remain in the atmosphere for a considerable period. Thus, depending on the location of the RCA’s origin, the aggregates may contain mercury. In the case of RCAB, the proximity to the sea and industrial zone means that the concrete had been exposed to the mercury present in the atmosphere and absorbed the mercury in its base matrix [34].

Another important indicator is iron (Fe) found in natural aggregates, and its value in RCA decreases to approximately 50%. This can be associated with the nature of the area where the natural aggregates were exploited and now as part of the RCA.

#### 3.2.4. Potential Reactions of RCAs in Asphalt Mixtures

This section seeks to analyze the chemical stability and possible interactions between the aggregates and the asphalt cement, assuming that these aggregates are used in the manufacture of asphalt mixtures.

It should be noted that the temperature at which the asphalt mixture works depends on environmental factors and the frequency and type of traffic once it begins its service. The only time where this mixture is exposed to high temperatures (above 150 °C) is during its manufacturing and storage. Likewise, the type of technology used in manufacturing the mixture (traditional or by emulsion) does not significantly affect the time because the system is subjected to high temperatures for a few hours [41]. The reactions that may take place to depend on several factors, among which the most important are temperature and reactant concentration [42,43].

The first reaction to be considered is the nitration of hydrocarbon compounds, which consists of the inclusion of a nitro group (NO_2_) to the hydrocarbon chain [44]. However, both a nitrating agent (nitric acid) and a catalyst (sulfuric acid) are needed for this reaction to occur. Although the presence of SO_3_ in the aggregates could indicate the presence of sulfuric acid, its concentration and nitric acid together are not sufficient to induce these reactions. Therefore, the only fixed nitrogen in the aggregates is in the form of nitrates (probably as sodium or potassium salts; see Table 7). These salts are stable compounds, which will not tend to react with the hydrocarbon. Additionally, as can be seen in Table 7, the nitrate content is in parts per million, which minimizes its possible reaction with the organic compounds. On the other hand, although the nitration reactions are exothermic [44], the conditions of the heavy hydrocarbon and asphalt mixture are such that there is no greater chemical potential (Gibbs free energy) for the nitration reaction to occur. Therefore, discard it among the series of reactions that this research intends to analyze.

The second reaction is the sulfonation of the compounds present in asphalt. This possible reaction consists of the inclusion of the sulfonic acid group (–SO_3_H), or the corresponding salt or halogen group of sulfonic acid (–SO_2_Cl) in an organic compound [44]. For this reaction to occur, the organic compound must contact a sulfonating agent, which may be sulfur trioxide, oleum, or concentrated sulfuric acid. In this case, the product formed would have amphiphilic properties, that is, a double affinity for both water and hydrocarbon, which could even improve the accession between the aggregate and asphalt [44]. However, the reaction with sulfur trioxide occurs in the gas phase; the sulfur present as SO_3_ indicates that there could be sulfuric acid present in the aggregates (Table 6), but this concentration in combination with the temperature conditions is not sufficient to stimulate the reaction. Therefore, although these types of reactions take place at low temperatures, they are conditioned to very high concentrations of sulfuric acid, which is a scenario far from the asphalt mixing systems. In this study, the SO_3_ content in the aggregates did not exceed 3% by weight of the samples, as can be seen in Table 6. Equally, the higher content of SO_3_ indicates that these types of reactions (with shallow conversion) could occur. Besides, the content of SO_3_ could even act as an adhesion-improving agent.

The third reaction considered in this analysis is the amination of the organic compounds present in the asphalt. This reaction involves the addition of the amine group by reducing nitro, nitroso, hydroxylamino, azoxy, azi, and hydrazo organic compounds. Amines may also be formed by reacting compounds containing certain labile groups (i.e., halogens, hydroxyl, and sulfonic) with ammonia [44]. Although the production of these compounds could improve the final adhesion between the aggregate and binder (in the same way as sulfonated surfactant compounds), these reactions are discarded in asphalt mixtures. The main reason is that there is no evidence of the presence or production of nitrated compounds (as discussed above), which makes impossible the amination reactions by reduction. Similarly to the nitration reactions, the fixed nitrogen in the system only exists as nitrates (salts), which are compounds that only ionize in aqueous solutions and are inert in a solid-state form when in contact with hydrocarbons.

Before considering the possible oxidation reactions, it should be noted that the halogenation reactions of the asphalt binder are not spontaneous under the temperature and concentration conditions where the mixture is found. In these reactions, halogen atoms are introduced into the hydrocarbon chain [44]. Although it is possible to determine the presence of halogens such as chlorine in the aggregates forming chlorides (Table 7), chlorine is found to form stable salts that are unreactive with the organic compounds present in the asphaltic binder. Likewise, as can be seen in Table 7, the chloride concentrations in the aggregates are in hundreds of parts per million for the recycled aggregates (RCAB and RCAP), whereas the level in the natural aggregate is in parts per million. The higher concentrations in the RCAs may be attributable to their exposure to environmental factors (atmospheric pollutants). However, in none of the cases can it be stated that they generated halogenation reactions with the binder compounds.

For the fourth reaction case, reactions could occur by alkali activation, which can improve the production of the binder and binder material. In Table 6, we find that the molar proportions do not suggest the generation of alkaline activation solutions, which require a molar ratio of 3.4 between SiO_2_ and Na_2_O [45]. In addition, there is no use of sodium hydroxide NaOH in the mixture as a reactive material that promotes the generation of alkaline activation solution. According to Figure 11 and Figure 12, the proportions of SiO_2_ and CaO (alkaline oxides) can be approximated in a molar proportion as the substitution percentage of the RCA (RCAP and RCAB) in the mixture increases. The proportion of SiO_2_ decreases while that of CaO increases when more RCA is added, without reaching the required values of molar proportion. There are aggregates with significant silicon and calcium contents such as quartz (SiO_2_) and calcite (CaCO_3_) in addition to other silicates (silico-aluminates) such as albite (NaAlSi_3_O_8_), muscovite KAl_2_(AlSi_3_O_10_) (OH)_2_, and anorthoclase (6SiO_2_Al_2_O_3_(K, Na)_2_O). These aggregate types have proportions lower than 5% and require generating the necessary conditions by adding other solutions to increase the molar amount of sodium species (Na) and promote alkaline reactions.

Finally, it is important to underline that even though in this work the evolution of the chemical composition of an asphalt mixture (using RCAs) was not monitored, some research allows us to affirm that the mixture is sufficiently stable over time. Asphalt binders have been used before as stabilization matrices for non-hazardous and hazardous wastes [46,47,48]. Besides, other recycled solid-asphalt mixtures have been developed, including hazardous solid effluents from the oil refining industry [49,50,51]. In this case, despite solids containing heavy metals, they remain stable in the asphalt matrix, preventing their escape through leaching into the environment [52]. These are some examples that allow us to conclude that the reactivity of the system over time can be neglected when using asphalt-RCAs mixtures.

## 4. Conclusions and Recommendations

Based on the results of the study, it can be concluded that:

- The physical characterization showed that the RCAs have lower density and higher absorption than NA. This result can be explained by the presence of mortar adhered in RCAs. As an RCA replaces the NA, the optimal asphalt content (OAC) increases due to higher absorption of the RCA, which ultimately increases the cost of the asphalt mixture.
- Coarser RCAs (25.0–19.0 to 9.5–4.75 mm) primarily contain quartz, calcite, and dolomite, which provide chemical and mineralogical characteristics suitable for use in asphalt mixtures.
- The increase in the concentration of CaO in the RCA, the decrease in SiO_2_ concerning the NA, and pH higher than 11.7, all promote the adhesion with the binder in the asphalt mixtures.
- The ratio of SiO_2_ to Na_2_O found in the RCAs, the reduction in the ratio between SiO_2_ and CaO (alkaline) in the replacements (15%, 30%, and 45%) of RCAs, and the absence of NaOH in non-asphalt mixtures promote alkaline activation reactions, which favor the chemical stability of the mixture.
- Although the exposure time of the RCAs to the coastal environment led to a high concentration of Cl^−^ ions—up to 482 mg/L, some of Cl^−^ ions came from stable salts that were not reactive with the organic compounds present in the asphalt binder.
- The nitrate and sulfate contents in RCAs do not promote nitration and sulfonation reactions owing to the absence of catalytic agents such as nitric and sulfuric acids, which favor chemical stability in asphalt mixtures.
- Dissolved metals in RCAs support the use of RCAs in asphalt mixtures because the lead and mercury contents do not generate adverse environmental impacts.
- Based on the chemical, mineralogical, and physical characteristics of the RCAs, the replacement of NA with an RCA in the manufacture of hot mix asphalt is an alternative viable. According to the results of this study, it is suggested to improve regulations to promote the use of RCAs in the manufacture of asphalt mixtures with less environmental impact in relation to conventional aggregates.

Despite having very good and interesting results in this research, the authors think that more experiments are necessary to obtain a more robust average of the results. For example, more analysis with different sources is necessary to obtain the RCAs and to understand the performance in the mixture. Other important laboratory experiment should analyze the absorption of the asphalt with different RCAs in order to assess the asphalt consumption and the costs of implementation in projects of roads. Future studies should also include the formulation of analysis guides for RCAs, where the way to chemically study them is standardized, and thus obtain indices and potential reactions between aggregates and asphalt mixtures, which should give standard data and reactions.

## Figures and Tables

**Table 1:** Summary of the literature review on the results of RCA physical properties tests.

Reference	Test				
	Specific Gravity Bulk (Fine Aggregate)	Specific Gravity Bulk (Coarse Aggregate)	LA Abrasion Test (%)	Absorption of Fine Aggregate, <4 mm (%)	Absorption of Coarse Aggregate (>4 mm) (%)
[26]	2.37	2.45~2.48	32~38	7.9	3.9~4.1
[27]	2.092	2.412	22	–	–
[28]	2.63	2.63	34	6.1	6.1
[29]	2.32	2.32	32	4.9	4.9
[30]	2.591	2.591	33.6	6.91	6.91
[31]	2.32	2.32	32.3	8.52	4.88
[32]	2.28	2.28	31	5.8	5.8

**Table 2:** Summary of the literature review on the results of the XRF test.

Reference	XRF Test									
	SiO_2_	CaO	Al_2_O_3_	Fe_2_O_3_	Na_2_O	MgO	SO_3_	K_2_O	TiO_2_	MnO
[33]	42.95–38.65	22.8–19.24	8.85–7.26	3.63–3.09	1.06–0.94	5.11–4.63	–	1.6–1.31	0.39–0.29	0.15–0.12
[8]	68.4	5.8	11.2	3.3	1.7	1	0.2	3.2	–	–
	68.6	6.5	10.2	3.3	1.6	1.2	0.3	2.8	–	–
	65.3	8.2	10.1	3.3	1.4	1.6	0.2	2.6	–	–
	65.6	8.8	9.3	3.2	0.9	1.2	0.2	2.7	–	–
[11]	58.29	13.27	7.69	6.12	1.45	2.28	0.92	0.8	0	0.16
[34]	62.56	12.01	12.52	5.82	2.69	1.83	–	1.3	0.62	0.12

**Table 3:** Distribution size of HMA-25 [36].

HMA-25	Sieve (mm)	25	19	12.5	9.5	4.75	2	0.425	0.18	0.075
% pass	Upper Limit	100	95	85	77	59	45	25	17	8
	Lower Limit	100	80	67	60	43	29	14	8	4

**Table 4:** Aggregate mixes.

Sieve (mm)	Fraction	100% NA	85% NA–15% RCA		70% NA–30% RCA		55% NA–45% RCA	
			NA %	RCA %	NA %	RCA %	NA %	RCA %
19	Coarse	14	11.9	2.1	9.8	4.2	7.7	6.3
12.5		17	14.5	2.6	11.9	5.1	9.4	7.7
9.5		8.5	7.2	1.3	6	2.6	4.7	3.8
4.75		10	8.5	1.5	7	3	5.5	4.5
NS	Fine	4	4	0	4	0	4	0
NWS		43	43		43		43	
Filler	–	3.5	3.5		3.5		3.5	

Note: NS = natural sand, NWS = natural washed sand.

**Table 5:** Specific gravity and water absorption of coarse NA, RCAP, and RCAB.

Sieve Size (mm)	NA		RCAP		RCAB	
	Specific Gravity	Water Absorption (%)	Specific Gravity	Water Absorption (%)	Specific Gravity	Water Absorption (%)
25.0–19.0	2.671	1.105	2.342	4.663	2.306	5.95
19.0–12.5	2.669	1.11	2.338	4.9	2.302	6.257
12.5–9.5	2.671	1.139	2.278	5.604	2.281	6.423
9.5–4.75	2.641	1.202	2.258	6.074	2.275	6.617

**Table 6:** XRF chemical compositions of NA, RCAP, and RCAB.

Main Constituents	(%)		
	NA	RCAB	RCAP
Na_2_O	5.45	7.1	7.1
MgO	2.73	5.73	4.85
Al_2_O_3_	12	8.65	7.85
SiO_2_	49.88	34.43	32.13
SO_3_	1.6	0.7	0.75
K_2_O	0.99	1.03	1.05
CaO	20.85	39.08	43.1
TiO_2_	0.33	0.16	0.13
MnO	0.17	0.05	0.05
Fe_2_O_3_	5.85	3.06	3.01

**Table 7:** Results of the physicochemical analysis (UV spectrophotometer).

Aggregate	Size (mm)	NO_3_^−^ (mg/L)	SO_4_^−2^ (mg/L)	Cl^−^ (mg/L)
NA	25.0–19.0	0.7	114	21
	19.0–12.5	1	113	135
	12.5–9.5	0.8	63	43.75
	9.5–4.75	1.4	43	35.75
RCAB	25.0–19.0	1.1	37	242
	19.0–12.5	0.8	33	166
	12.5–9.5	0.9	49	153
	9.5–4.75	0.9	45	154
RCAP	25.0–19.0	3.13	16	482.5
	19.0–12.5	1.6	31	217
	12.5–9.5	1	33	269
	9.5–4.75	0.8	44	213

**Table 8:** Atomic absorption spectrometry pf water-soluble elemental contents (mg/L).

Aggregate	Size (mm)	Fe	Al	Si	Ti	Ca	Pb	Zn	Ni	Mg	Mn	Hg
NA	25.0–19.0	135.5	13.9	1777	1.89	172.9	<0.3	0.3	<0.1	16.9	5.2	–
	19.0–12.5	173.2	16.2	1966	1.18	230.2	<0.3	0.4	<0.1	17.1	5.7	<10
	12.5–9.5	184.5	15.2	2008	1.18	177.6	<0.3	0.4	<0.1	11	5.3	<10
	9.5–4.75	169.8	16.5	2460	1.09	139.2	<0.3	0.3	<0.1	9.32	5.1	<10
RCAB	25.0–19.0	54.62	15.9	854.4	1.45	314.7	<0.3	0.2	<0.1	62.1	1.2	<10
	19.0–12.5	60.87	17.8	1886	1.18	317.4	<0.3	0.2	<0.1	61.3	1.5	<10
	12.5–9.5	46.48	18.6	1476	1.36	327.5	<0.3	0.2	<0.1	40.2	1.6	<10
	9.5–4.75	73.09	19.8	1415	<1.00	389.8	<0.3	0.3	<0.1	38.8	1.5	11.6
RCAP	25.0–19.0	73.31	22.4	1142	<1.00	359.5	<0.3	0.2	<0.1	54.5	1.5	<10
	19.0–12.5	61.38	13	1236	<1.00	320.2	<0.3	0.1	<0.1	45.1	1.6	<10
	12.5–9.5	53.38	14.2	1123	1.8	284.7	<0.3	0.1	<0.1	39.8	1.4	<10
	9.5–4.75	54.04	10.8	1316	<1.00	305.5	<0.3	0.1	<0.1	36	1.3	<10
